# Fetal haemoglobin and bronchopulmonary dysplasia in neonates: an observational study

**DOI:** 10.1136/archdischild-2020-319181

**Published:** 2020-08-26

**Authors:** William Hellström, Tobias Martinsson, Ann Hellstrom, Eva Morsing, David Ley

**Affiliations:** 1 Department of Pediatrics, University of Gothenburg, Institute of Clinical Sciences, Gothenburg, Sweden; 2 Department of Clinical Sciences Lund, Pediatrics, Lund University, Skåne University Hospital, Lund, Sweden; 3 Department of Ophthalmology, University of Gothenburg, Institute of Neuroscience and Physiology, Gothenburg, Sweden

**Keywords:** neonatology, physiology, pathology

## Abstract

**Objective:**

Early decrease in fetal haemoglobin (HbF) is an indicator of loss of endogenous blood components that might have predictive value for development of bronchopulmonary dysplasia (BPD). The link between HbF and BPD has not been evaluated.

**Design:**

Retrospective observational study.

**Setting:**

Tertiary level neonatal intensive care unit, referral centre for Southern Sweden.

**Patients:**

452 very preterm infants (<30 gestational weeks) born 2009–2015.

**Interventions:**

Regular clinical practice.

**Main outcome measures:**

Mean HbF, haemoglobin (Hb) and partial oxygen pressure (PaO_2_) levels calculated from 11 861 arterial blood gas analyses postnatal week 1. Relationship between HbF (%) and BPD (requirement of supplemental oxygen at 36 weeks’ postmenstrual age) and the modifying influence of PaO_2_ (kPa) and total Hb (g/L) was evaluated.

**Results:**

The mean gestational age (GA) at birth was 26.4 weeks, and 213 (56%) infants developed BPD. A 10% increase in HbF was associated with a decreased prevalence of BPD, OR 0.64 (95% CI 0.49 to 0.83; p<0.001). This association remained when adjusting for mean PaO_2_ and Hb. Infants with an HbF in the lowest quartile had an OR of 27.1 (95% CI 11.6 to 63.4; p<0.001) for development of BPD as compared with those in the highest quartile. The area under the curve for HbF levels and development of BPD in the full statistical model was 0.871.

**Conclusions:**

Early rapid postnatal decline in HbF levels was associated with development of BPD in very preterm infants. The association between HbF and BPD was not mediated by increased oxygen exposure. The potential benefit of minimising loss of endogenous blood components on BPD outcome will be investigated in a multicentre randomised trial.

**What is already known on this topic?**

- Bronchopulmonary dysplasia (BPD) is a major, chronic oxygen-related lung disease affecting preterm infants.
- During the first weeks of life, a majority of the endogenous blood containing many important fetal components is exchanged for adult blood due to clinical intervention.

**What this study adds?**

- This is, to our knowledge, the first time the link between low fetal haemoglobin (HbF) levels and the development of BPD has been shown.
- Minimising early postnatal loss of endogenous blood components and thus maintaining levels of HbF may be essential for the prevention of BPD in the very preterm infant.
- The impact of maintaining HbF levels and the link to BPD is currently being investigated in an ongoing randomised multicentre trial.

## Introduction

The blood of the healthy fetus contains cellular and molecular components essential for normal development.^1^ Very preterm transition from fetal to postnatal life involves an immediate disconnection from placento-maternal support, and the continued postnatal production of endogenous factors is influenced by an environment different from that in utero. Substantial differences in nutritional intake, exposure to relative hyperoxia and exogenous stressors inducing proinflammatory reactions collectively inhibit endogenous synthesis of trophic factors and have all been related to subsequent morbidity.^2–4^

The depletion of endogenous factors associated with very preterm birth is considerably amplified by interventions routinely performed in neonatal intensive care. We have recently shown that blood sampling in extremely preterm infants during the first two postnatal weeks resulted in an iatrogenic blood loss of, on average, 40 mL/kg, corresponding to 58% of the circulating blood volume. This iatrogenic depletion of blood correlated with the volume of transfused adult donor blood. Of note, sample-related blood volume loss during the first postnatal week was associated with development of bronchopulmonary dysplasia (BPD).^5^

In newborn infants, fetal haemoglobin (HbF) constitutes the majority of haemoglobin (Hb). Normally, the proportion of HbF decreases very gradually as the endogenous production of adult haemoglobin proceeds, a process completed within the first 25 weeks following birth.^6^ In the very preterm infant, the proportion of HbF during the first postnatal weeks will thus serve as a sensitive quantitative marker of iatrogenic blood loss compensated by transfusions with adult donor blood leading to an exchange of HbF with adult haemoglobin (HbA). The relationship between administered blood transfusions and decreased levels of HbF was shown recently in a relatively small cohort of very preterm infants. An underlying mechanism was proposed involving a shift of the oxygen dissociation curve due to replacement of HbF by HbA, resulting in a higher fraction of oxygen dissolved in plasma and an increased exposure of tissue to oxygen.^7^

We hypothesised that early postnatal decrease in HbF would serve as a biomarker for development of BPD in very preterm infants. This was evaluated in 452 consecutively admitted inborn infants. Levels of HbF were evaluated together with concomitant measures of arterial oxygen tension (PaO_2_) and of total Hb in order to discern possible effects of decreased HbF induced by increased exposure of tissue to dissolved oxygen as well as effects mediated by anaemia.

## Material and Methods

### Patients and clinical characteristics

This single-centre retrospective observational study included infants (n=452) live-born before 30 weeks of gestational age (GA), delivered at Skåne University Hospital in Lund and admitted to the tertiary level neonatal intensive care unit (NICU) in Lund between 2009 and 2015. Nine infants were lost to follow-up at postmenstrual age (PMA) 36 weeks due to hospital transfer, two infants with available BPD data (no BPD) and three infants without BPD data (deceased prior to 36 weeks’ PMA) had incomplete longitudinal blood gas variable data during postnatal days (PND) 1–7 and thus were not included in the HbF, PaO_2_ and Hb analyses. GA was determined by routine ultrasound fetometry at 17–18 postmenstrual weeks. Birth weight (BW) small for gestational age (SGA) was defined as a BW >2 SD below the GA-related mean of the population according to the Swedish reference curve for normal fetal growth.^8^ During this time period, infants, when needed, received supplemental oxygen aiming to achieve a peripheral oxygen saturation (SpO_2_) of 91%–95%. Blood transfusions were administered to maintain Hb concentrations above 140 g/L during the first postnatal week. BPD was defined as a requirement of supplemental oxygen at 36 weeks’ postmenstrual age. The diagnosis of necrotising enterocolitis (NEC) was based on clinical and radiographic findings showing severe abdominal distension in combination with radiographic detection of intramural or intrahepatic gas or signs of intestinal perforation (modified Bell criteria). Clinical Risk Index for Babies scoring system II (CRIB-II score) was individually calculated based on GA at birth, BW, body temperature at admission and base excess the first 12 hours.^9^ Ninety-eight of the infants were included as a subcohort in a previous bicentre study in Sweden when examining sampling-related blood volumes and blood transfusions.^5^

### Blood gas analyses

In total, 11 861 arterial blood gas measurements were obtained and analysed during the first 7 PNDs. Non-arterial (venous and capillary) blood gas analyses were excluded from the study. Blood gas data were retrieved from the local hard drive of the blood gas analyser (Radiometer 800, Copenhagen, Denmark) in the NICU. Variables included in the analysis were: PaO_2_ (kPa), total Hb (g/L) and HbF (%). Blood gas analyses from each individual were sorted according to time-point and date of analysis. Blood gas variables per individual were then averaged for each PND (PND 1–7). For each blood gas variable, the mean value for the first postnatal week was assessed in data analysis as a representation of blood gas variables, as it was based on a solid and similarly distributed foundation of aggregated data.

### Statistics

The data were analysed using IBM SPSS V.25 and SAS software V.9.4. All assumptions for logistic regression were fulfilled for included variables. Regression models assessed in the statistical analyses were verified in terms of multicollinearity; variance inflation factor (VIF) score >1, <10, independent observations and outliers; Cook’s distance <1 and leverage below a threshold level of <3 times (k+1)/N. If outlier was present, it was excluded and models rerun. Rerun analyses yielding non-similar results as compared with original model were displayed if present. The Hosmer and Lemeshow test was assessed for evaluating goodness of fit and was required to be non-significant. The mean of each blood gas variable during postnatal week 1 was used in logistic regression analysis. CRIB-II score was coded as high (CRIB-II ≥11, corresponding to CRIB level 3–4) or low (CRIB-II level <11, corresponding to CRIB level 1–2).^9^ Independent confounding variables included in the full model were GA at birth, BW SGA, sex, highest and lowest fractional inspired oxygen (FiO_2_) during the first 12 hours of life and CRIB-II score (dichotomous). The independent respective contributions of HbF, PaO_2_ and total Hb to BPD during postnatal week 1 were assessed using multiple variable analysis. Independent predictive abilities were evaluated with area under the curve (AUC) values using receiver operating characteristic (ROC) analysis. Predicted probabilities were obtained by using logistic regression. In all analyses, p values <0.05 were considered significant.

## Results

**Figure 1:**
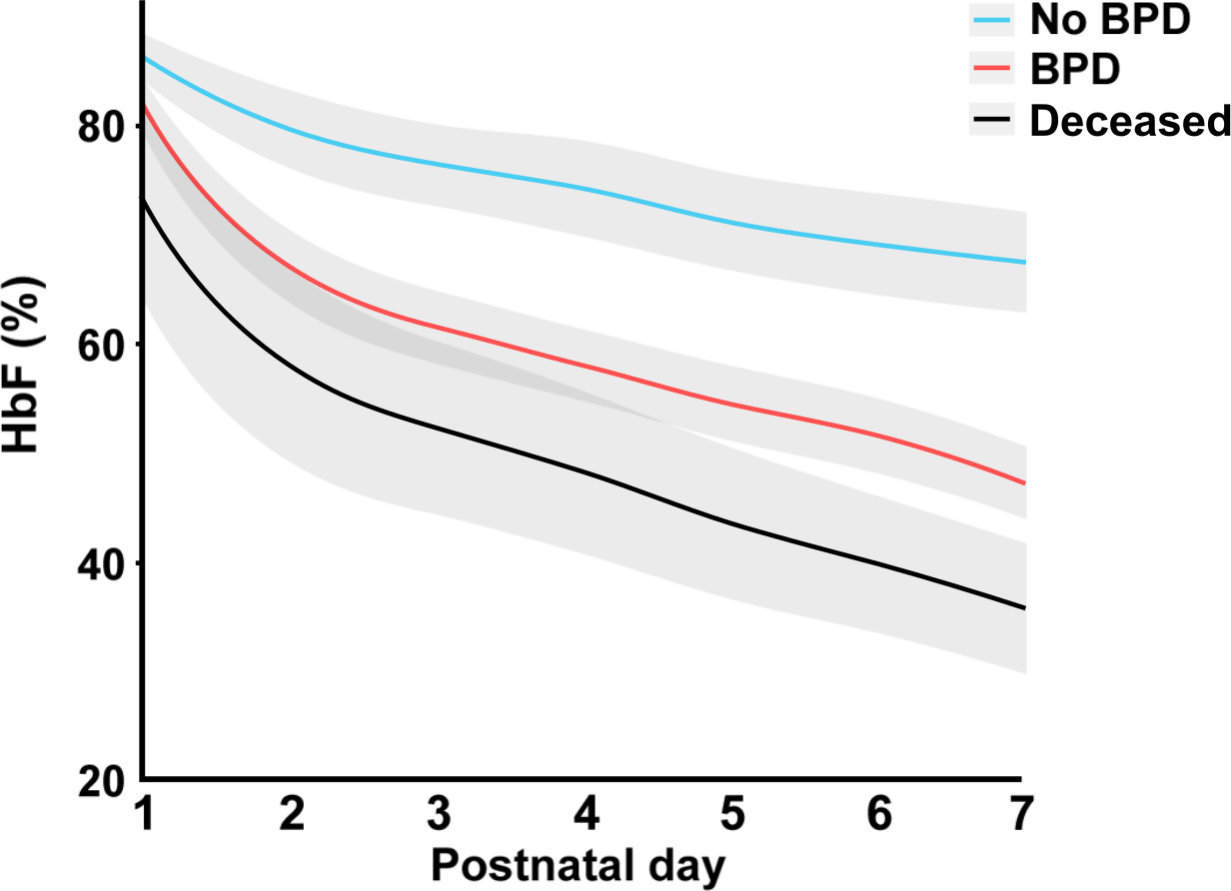
Mean levels (%) of HbF during postnatal days 1–7 in relation to development of BPD and non-survival. Infants with no BPD (n=169 in the complete cohort)=light blue; infants with BPD (n=213)=red; infants who died prior to PMA 36 weeks (n=61 in the complete cohort)=black. The shadowed area illustrates a 95% CI. Figure was generated based on an aggregated daily mean, and an interpolation curve was generated connecting daily data. BPD, bronchopulmonary dysplasia; HbF, fetal haemoglobin; PMA, postmenstrual age.

During the study period, 452 live-born infants were delivered, with a mean (SD) GA of 26.4 (1.8) weeks and a mean (SD) BW of 876 (272) g. The overall mortality rate prior to 36 weeks’ PMA was 61/452 (13.5%). In total, 213 (55.8%) infants developed BPD. Data on clinical characteristics are given in table 1. The number of blood gases available for analysis on PND 1–7 was 1386, 2389, 2112, 1773, 1523, 1366 and 1312, respectively. The median (IQR) number of blood gases per subject and PND on PND 1–7 were 3 (2–4), 5 (3–7), 4 (3–7), 3 (2–6), 3 (1–5), 3 (1–5) and 3(1–5). Mean values of HbF (%) during the first postnatal week in relation to postnatal age (days) in infants with and without BPD and in non-surviving infants are displayed in figure 1. Respective relationships between mean HbF (%), total Hb (g/L) and PaO_2_ (kPa) during postnatal week 1 and development of BPD are shown in table 2. Infants with BPD displayed significantly lower mean levels of HbF (%), Hb (g/L) and total PaO_2_ (kPa) during the first postnatal week, before and after adjustment for GA at birth, BW SGA, sex, highest and lowest FiO_2_ (12 hours) and CRIB-II score, compared with infants without BPD. When combining infants with BPD and infants deceased before 36 weeks’ PMA into a composite outcome variable, a 10% increase in mean HbF (%) in the full statistical model was associated with a decrease in the composite outcome (OR of 0.63 (95% CI 0.48 to 0.82, p<0.001)).

**Table 1:** Clinical characteristics and blood gases per subject PND 1–7

	Total n=452
GA, weeks; mean (SD)	26.4 (1.8)
Birth weight, g; mean (SD)	876 (272)
BW SGA; n (%)	111 (24.6)
Mortality*; n (%)	61 (13.5)
Male; n (%)	260 (57.5)
Twins; n (%)	110 (24.3)
Triplets; n (%)	18 (4)
**Morbidities**	
IVH grades 3–4; n (%)†	43 (9.9)
Any IVH; n (%)†	119 (27.4)
NEC; n (%)‡	23 (5.4)
BPD; n (%)§	213 (55.8)
CRIB-II score; mean (SD)¶	11.4 (3.3)

*Before 36 weeks’ PMA.

†435 available for analysis.

‡425 available for analysis.

§382 available for analysis.

¶212 available for analysis.

BPD, bronchopulmonary dysplasia; BW SGA, birth weight small for gestational age; CRIB-II, Clinical Risk Index for Babies scoring system II; GA, gestational age; IVH, intraventricular haemorrhage; n, number; NEC, necrotising enterocolitis; PMA, postmenstrual age; PND, postnatal day.;

**Table 2:** Distribution of HbF, Hb and PaO_2_ during postnatal week 1 in relation to BPD

		BPD		Unadjusted		Adjusted*		Adjusted†	
**Postnatal week 1**		**Yes**	**No**	**P value**	**OR by a 95% CI**	**P value**	**OR by a 95% CI**	**P value**	**OR by a 95% CI**
HbF	Mean % (SD)	61.8 (19.1)	78.6 (14.7)	**<0.001**	0.55 (0.47 to 0.64)‡	**<0.001**	0.63 (0.52 to 0.77)‡	**<0.001**	0.64 (0.49 to 0.83)‡
Hb	Mean g/L (SD)	146.3 (8.6)	153.7 (11.3)	**<0.001**	0.44 (0.34 to 0.57)‡	**0.002**	0.62 (0.46 to 0.84)‡	**<0.001**	0.36 (0.20 to 0.64)‡
PaO_2_	Mean kPa (SD)	7.4 (0.7)	8.2 (1.3)	**<0.001**	0.47 (0.36 to 0.59)	**<0.001**	0.43 (0.30 to 0.60)	**0.004**	0.45 (0.26 to 0.77)

*Adjusted for gestational age at birth, birth weight small for gestational age, sex, highest and lowest FiO_2_ (12 hours).

†In the full statistical model, adjusted for gestational age at birth, birth weight small for gestational age, sex, highest and lowest FiO_2_ (12 hours) and CRIB-II score.

‡OR by a 10-unit increase p values <0.05 in bold.

BPD, bronchopulmonary dysplasia; CRIB, Clinical Risk Index for Babies scoring system II; FiO_2_, fractional inspired oxygen; Hb, haemoglobin; HbF, fetal haemoglobin; PaO_2_, partial oxygen pressure.

Mean levels of HbF (%) remained significantly associated with BPD in the full statistical model after adjustment for mean PaO_2_ (kPa) and mean Hb (g/L): a 10% increase in HbF was associated with a decreased rate of BPD, OR of 0.72 (95% CI 0.54 to 0.97, p=0.032).

**Figure 2:**
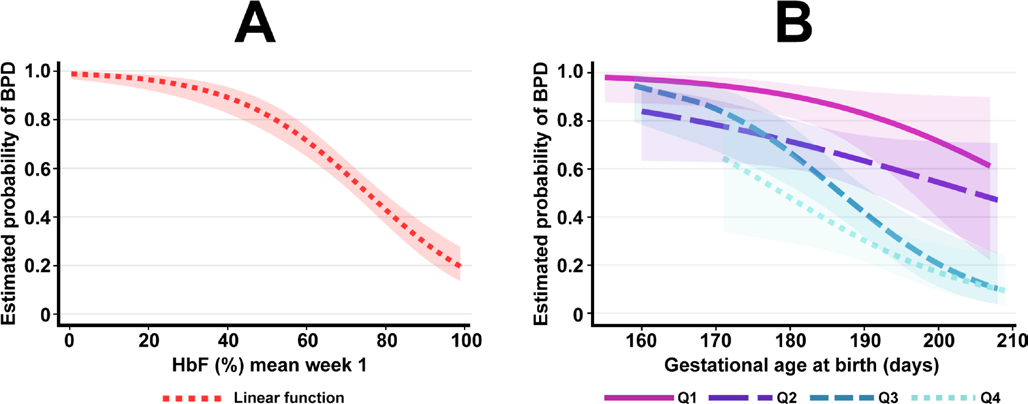
(A and B) Estimated probability for BPD in relation to HbF (%) and GA at birth. Illustrated by linear function by mean HbF (%) in postnatal week 1 in the complete cohort (A) and by GA at birth illustrated per quartile of HbF in postnatal week 1 (B). Q1 represents the lowest quartile of mean HbF in postnatal week 1 in B, where Q4 represents the highest proportional values of HbF. Q4: >77.6%, Q3: 74%–77.6%, Q2: 58.8%–73.9% and Q1: <58.8% HbF. The profiles of Q2 and Q3 do not follow proportional odds across the studied material. In total, 11 861 blood gases were analysed in a cohort of 452 consecutively born infants. The shadowed area illustrates a 95% CI. BPD, bronchopulmonary dysplasia; GA, gestational age; HbF, fetal haemoglobin; Q, quartile.

Infants with a mean HbF (%) in the lowest quartile during the first postnatal week had an OR of 27.1 (95% CI 11.6 to 63.4; p<0.001) for development of BPD when compared with those with an HbF (%) in the highest quartile. Estimated probability plot for BPD for mean HbF (%) and for each quartile of HbF (%) during postnatal week 1 considering GA at birth are illustrated in figure 2A–B. The profiles of quartile 2 and quartile 3 did not follow proportional odds across the studied range of GA.

**Figure 3:**
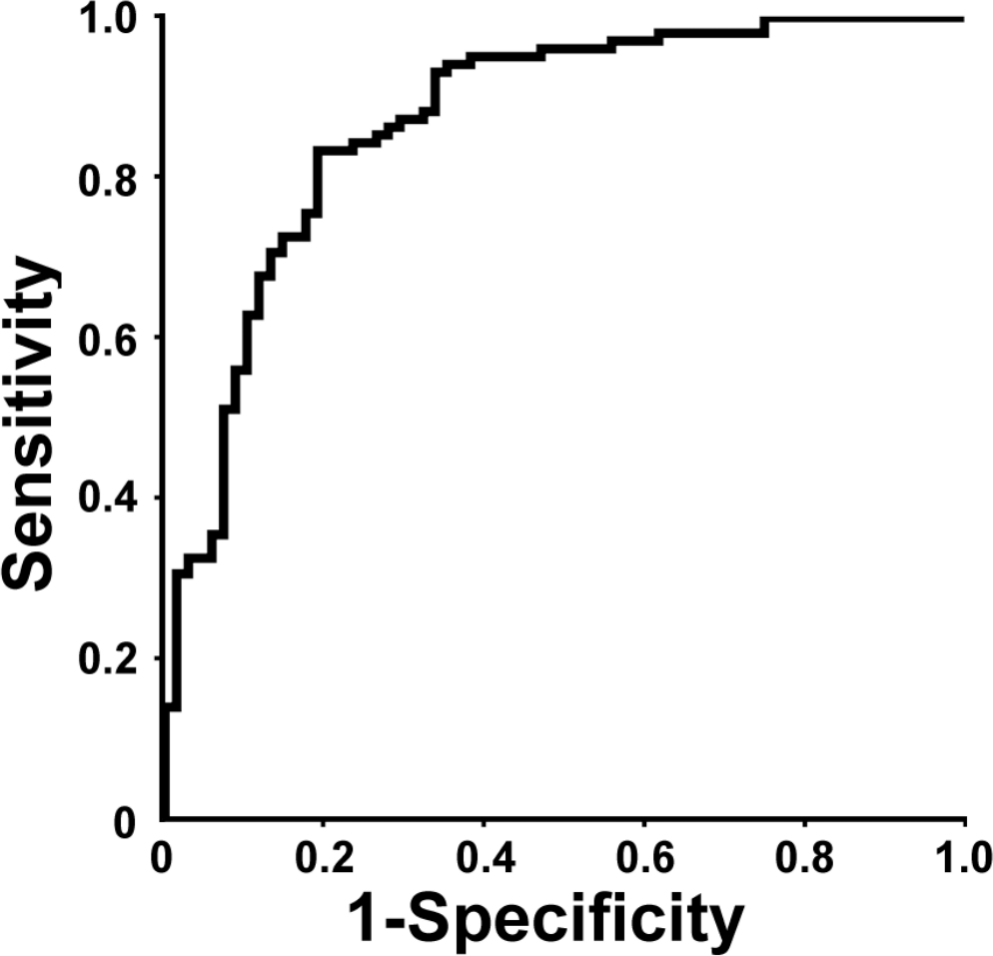
ROC curve for BPD and adjusted levels of HbF (%) during postnatal week 1. HbF (%) adjusted for GA at birth, BW SGA, sex, highest and lowest FiO_2_ and CRIB-II score in the ROC curve. The area under the curve was 0.871 in the full statistical model. A total of 11 861 blood gases were analysed in a cohort of 452 consecutively born infants. BPD, bronchopulmonary dysplasia; BW SGA, birth weight small for gestational age; CRIB-II, Clinical Risk Index for Babies scoring system II; FiO_2_, fraction of inspired oxygen; GA, gestational age; HbF, fetal haemoglobin.

The AUC of baseline parameters (GA at birth, sex, BW SGA, high and low FiO_2_ and CRIB-II score) for prediction of BPD was 0.841. When mean HbF (%) during the first postnatal week was added to the model, the AUC increased to 0.871 (figure 3). The AUC for mean PaO_2_ and Hb in the full model were 0.861 and 0.869, respectively.

## Discussion

This study is, to our knowledge, the first to show that decreased levels of HbF during early postnatal life are robustly associated with the development of BPD in very preterm infants. Decreased HbF levels are a direct consequence of sample-related neonatal blood volume loss compensated for by transfusions with adult donor blood containing HbA. This finding emphasises the importance of developing and implementing blood saving sampling methodology in the clinical care of very preterm infants.

The significant decrease in HbF observed during the first postnatal week reflects current blood sampling strategy and existing laboratory infrastructure for blood sample analysis in this very preterm population and corresponds well to our previously reported findings on sample-induced blood volume loss and transfused blood volume during the first 2 weeks of life.^5^ The changes in HbF levels were considerable, with a mean HbF for the whole cohort on PND 1 of 83% decreasing to a mean of 53% at PND 7. The difference in mean levels of HbF between infants who did or did not develop BPD was pronounced during the first PNDs and reached a maximum at 7 days. The probability of BPD was inversely related to mean HbF during the first postnatal week, with a probability of 20% for development of BPD at a mean HbF of 80% and a 90% probability at a mean HbF of 40%. The potential causal mechanisms involved in BPD development reflected by changes in HbF would thus appear to be operating during early postnatal development.

We have previously reported that weight-related blood sample volume loss and weight-related blood transfusion volume both increase with decreasing GA at birth.^5^ Importantly, the relationship between changes in mean HbF and development of BPD reported here was independent of GA at birth and other potential confounders studied. The probability for development of BPD also differed for the respective HbF quartiles across the range of GA at birth.

The causal mechanisms involved in BPD development reflected by a decrease in HbF are unknown but might be several. The postnatal proportion of HbF is inversely related to the accumulated volume of administered blood from adult donors. An increased proportion of HbA will have a direct effect on the dissociation curve of oxygen, resulting in a higher level of dissolved oxygen in plasma for a given oxygen saturation of Hb. Increased tissue exposure to oxygen has often been proposed as a contributor to several morbidities in very preterm infants, including BPD. However, the present data convincingly negate the hypothesis that the observed effect of decreased HbF levels on BPD was mediated by increased oxygen exposure, because: (1) the effect of HbF on BPD was independent of changes in PaO_2_ and (2) when both variables were evaluated in the same regression model, decreased HbF and PaO_2_ levels were, independently from each other, associated with development of BPD. In addition, higher levels of HbF have been shown to have beneficial oxygen-related effects in other organ systems, such as increased oxygen transport in the brain, due to the specific oxygen-carrying properties of HbF.^10^

As previously mentioned, decreased HbF levels are highly correlated with the volume of administered blood from adult donors. Several studies have shown associations between administered blood transfusions and increased rates of neonatal morbidity.^11 12^ However, prospective studies comparing liberal or restrictive transfusion strategies based on different Hb thresholds have not, as yet, been able to show a clear impact on either short-term or long-term morbidity.^13^

We speculate that the prime cause of morbidity reflected by changes in HbF is the loss of endogenous blood components essential for normal organ development. We have previously shown that circulating levels of the fetal growth factor insulin-like growth factor 1 (IGF-1) decrease abruptly during transition from fetal to postnatal life, and persisting low levels have been associated with development of BPD, retinopathy of prematurity (ROP) and restricted postnatal growth.^4^ Indeed, a deficiency of circulating IGF-1 in extremely preterm infants has been proposed as a basis for supplementary treatment, and results from a recent intervention trial suggested that treatment with the complex IGF-1/IGF-binding protein-3 decreased the severity of BPD.^14^ A significant reduction in iatrogenic blood loss in very preterm infants might have a significant impact on levels of circulating growth factors, thereby improving the outcome of these infants.^15^

The blood of the very preterm infant also contains unique cellular components of importance for development. The concentration of haematopoietic stem cells is inversely related to GA at birth.^1^ The beneficial effects of delayed cord clamping and the resulting autotransfusion of fetal blood on neonatal morbidities in preterm infants lends further support to the importance of maintaining, and not depleting, essential endogenous blood components, as recently demonstrated in a review.^16^

The main limitation of this study is the retrospective design. The presented data reflect care provided at a single Swedish level III NICU during a 7-year period. All consecutively admitted infants below 30 gestational weeks (GW) at birth were included in the study. The sampling procedures and sampled blood volumes at the NICU in Lund in relation to performed laboratory analyses have recently been reported in a subcohort of the present study population.^5^ The main strengths of this study are the size of the study cohort and the high number of obtained blood gas analyses rendering mean values for the first postnatal week based on high-resolution data. All blood gases measured in the present study were analysed on the same blood gas analyser, and blood gas data were downloaded directly from the analyzer’s hard disk, thereby minimising the risk of errors.

## Conclusion

Decreased levels of HbF during the first postnatal week were associated with the development of BPD in the very preterm infant. Conversely, maintaining a higher percentage of HbF may be protective for developing BPD. The association between HbF and BPD was not mediated by an increase in oxygen exposure. The potential beneficial role of minimising the loss of endogenous blood components in a clinical setting will be further investigated in an ongoing multicentre randomised trial.

## References

[1] Podestà M, Bruschettini M, Cossu C, et al Preterm cord blood contains a higher proportion of immature hematopoietic progenitors compared to term samples. PLoS One 2015;10:e0138680. 10.1371/journal.pone.0138680 26417990 PMC4587939

[2] Price WA, Lee E, Maynor A, et al Relation between serum insulinlike growth factor-1, insulinlike growth factor binding protein-2, and insulinlike growth factor binding protein-3 and nutritional intake in premature infants with bronchopulmonary dysplasia. J Pediatr Gastroenterol Nutr 2001;32:542–9. 10.1097/00005176-200105000-00010 11429514

[3] Pierce EA, Foley ED, Smith LE Regulation of vascular endothelial growth factor by oxygen in a model of retinopathy of prematurity. Arch Ophthalmol 1996;114:1219–28. 10.1001/archopht.1996.01100140419009 8859081

[4] Hellström A, Ley D, Hansen-Pupp I, et al Insulin-Like growth factor 1 has multisystem effects on foetal and preterm infant development. Acta Paediatr 2016;105:576–86. 10.1111/apa.13350 26833743 PMC5069563

[5] Hellström W, Forssell L, Morsing E, et al Neonatal clinical blood sampling led to major blood loss and was associated with bronchopulmonary dysplasia. Acta Paediatr 2020;109:679-687. 10.1111/apa.15003 31505053 PMC7155086

[6] Terrenato L, Bertilaccio C, Spinelli P, et al The switch from haemoglobin F to a: the time course of qualitative and quantitative variations of haemoglobins after birth. Br J Haematol 1981;47:31–41. 10.1111/j.1365-2141.1981.tb02759.x 6159914

[7] Stutchfield CJ, Jain A, Odd D, et al Foetal haemoglobin, blood transfusion, and retinopathy of prematurity in very preterm infants: a pilot prospective cohort study. Eye 2017;31:1451–5. 10.1038/eye.2017.76 28548651 PMC5639193

[8] Maršál K, Persson P-H, Larsen T, et al Intrauterine growth curves based on ultrasonically estimated foetal weights. Acta Paediatr 1996;85:843–8. 10.1111/j.1651-2227.1996.tb14164.x 8819552

[9] Ezz-Eldin ZM, Hamid TAA, Youssef MRL, et al Clinical risk index for babies (CRIB II) scoring system in prediction of mortality in premature babies. J Clin Diagn Res 2015;9:SC08–11. 10.7860/JCDR/2015/12248.6012 PMC4525567 26266178

[10] Ramaekers VT, Daniels H, Casaer P Brain oxygen transport related to levels of fetal haemoglobin in stable preterm infants. J Dev Physiol 1992;17:209–13. 1281182

[11] Wan-Huen P, Bateman D, Shapiro DM, et al Packed red blood cell transfusion is an independent risk factor for necrotizing enterocolitis in premature infants. J Perinatol 2013;33:786–90. 10.1038/jp.2013.60 23702619

[12] Baer VL, Lambert DK, Henry E, et al Among very-low-birth-weight neonates is red blood cell transfusion an independent risk factor for subsequently developing a severe intraventricular hemorrhage? Transfusion 2011;51:1170–8. 10.1111/j.1537-2995.2010.02980.x 21166684

[13] Scott PH, Berger HM, Kenward C, et al Effect of gestational age and intrauterine nutrition on plasma transferrin and iron in the newborn. Arch Dis Child 1975;50:796–8. 10.1136/adc.50.10.796 1236569 PMC1545688

[14] Ley D, Hallberg B, Hansen-Pupp I, et al rhIGF-1/rhIGFBP-3 in preterm infants: a phase 2 randomized controlled trial. J Pediatr 2019;206:56–65. 10.1016/j.jpeds.2018.10.033 30471715 PMC6389415

[15] Katsimpardi L, Litterman NK, Schein PA, et al Vascular and neurogenic rejuvenation of the aging mouse brain by young systemic factors. Science 2014;344:630–4. 10.1126/science.1251141 24797482 PMC4123747

[16] Lodha A, Shah PS, Soraisham AS, et al Association of deferred vs immediate cord clamping with severe neurological injury and survival in extremely Low-Gestational-Age neonates. JAMA Netw Open 2019;2:e191286–e. 10.1001/jamanetworkopen.2019.1286 30924898 PMC6450317

